# A new algorithm for the transconjunctival correction of moderate to severe upper eyelid ptosis in adults

**DOI:** 10.1038/s41598-024-52990-9

**Published:** 2024-01-31

**Authors:** Izabela Nowak-Gospodarowicz, Aleksandra Kicińska, Michał Kinasz, Marek Rękas

**Affiliations:** Department of Ophthalmology, Military Institute of Medicine-National Research Institute, 128 Szaserow St, 04-141 Warsaw, Poland

**Keywords:** Health care, Medical research

## Abstract

A posterior approach is recommended for the correction of mild to moderate upper eyelid ptosis in adults. The aim of this study is to propose a new algorithm that helps to predict outcomes in the transconjunctival correction of moderate to severe blepharoptosis. This study included adult patients with moderate to severe upper eyelid ptosis treated between 2019 and 2021. Patients meeting inclusion criteria underwent ptosis correction through a posterior approach using an algorithm: 4 mm Mueller’s muscle transconjunctival resection to correct 1 mm ptosis (depending on a test with 10% phenylephrine: 3–12 mm) ± tarsal plate resection: 1 mm for every 1 mm of residual ptosis after phenylephrine test, but leaving a minimum of 4 mm upper tarsus intact. Outcomes were ovserved within at least 6-months. Outcomes were assessed based on pre- and postoperative MRD1 changes, inter-eyelid height symmetry, cosmetic effect, and complications. Outcomes of 118 procedures in 81 patients (average age 69, range: 47–87) were analyzed. MRD1 changes were statistically significant, from 0.2 ± 1.6 mm before to 4.1 ± 1 mm after surgery. The function of the levator palpebrae superioris muscle was 10.2 ± 3.4 (range 5–17) mm. Upper eyelid lifted by an average of 1.8 ± 0.7 (range 0–3) mm after the instillation of 10% phenylephrine eyedrops. An average of 8.5 ± 0.8 (range 8–10) mm of conjunctiva and Mueller’s muscle and 2.2 ± 0.9 (range 1–5) mm of the tarsal plate were resected during the procedure. Inter-eyelid height symmetry within 1 mm was achieved in 95% of outcomes. The algorithm introduced in this study appears to be useful to achieve repeatable satisfactory outcomes in the transconjunctival correction of moderate to severe upper eyelid ptosis in adults with at least ”fair” levator function.

## Introduction

Upper eyelid ptosis is the incorrectly low position of the upper eyelid, which results in a limited visual field and the deterioration of visual acuity^1–4^. Effective treatment of this condition is an essential part of ophthalmologists’ practice. There are many methods of surgical correction of eyelid ptosis. Clinically, depending on the degree of the ptosis and anatomical condition, methods can be divided into those performed via the skin—externally and internally—via transconjunctival approach^5–7^**.** Transconjunctival methods, which do not leave a visible skin scar, have the additional advantages of shorter surgery time, faster reconvalescence and shorter surgeon’s learning curve^8,9^. The main reported disadvantage of transconjunctival methods is their limited efficacy to correct mild upper eyelid ptosis – less than 3 mm, preferably with a good function of the levator muscle^10,11^. There is also a lack of a single, reliable algorithm that predicts the exact outcomes of surgery based on the degree of ptosis and anatomical conditions (which vary among individuals)^8–13^. In 2002 Perry et al. suggest combining tranconjunctival resection of the Mueller’s muscle with “x “ amount of the tarsus to correct residual ptosis after a preoperative phenylephrine test with excellent results, but limited to patients with at least good levator muscle function and a maximum of 2.5 mm upper tarsus resection^13^. The anatomical studies of Goold et al. and Coban et al. reveal that the average upper tarsal plate height in Caucasians is 10.1 mm and 10.6 mm respectvely. Research has shown that 3 mm of tarsus is enough for stability of the upper eyelid^14,15^. Performing reconstruction of large eyelid defects after removal of eyelid malignant tumors with the use of a modified Hughes’flap and harvesting free tarsal grafts from the upper eyelids, we noticed that leaving at least 3.5–4 mm of the upper eyelid tarsus intact usually allows for the safe removal of approx. 3–5 mm without causing any instability of the upper eyelid^16,17^. We seek to combine reports of other authors about transconjunctival correction of the upper eyelid ptosis^10–13,18–26^ with anatomical studies^14,15,27,28^ and our remarks in order to: (1) evaluate the use of a modified transconjunctival technique for the correction of not only mild but also moderate to severe upper eyelid ptosis, and (2) establish an algorithm for estimating the amount of upper eyelid tissue required for resection depending on the degree of upper eyelid ptosis, anatomical conditions and the results of preoperative diagnostic tests.

## Method

### Study design

This was a prospective, interventional study that assessed clinical outcomes in patients after surgical correction of moderate to severe upper eyelid ptosis using a posterior (transconjunctival) approach between June 2019 and July 2021. The study was approved by the designated ethics committee (Bioethical Commission at the Military Medical Chamber in Warsaw; ethical approval number 238/22, received on 29 July 2022, administrative process having been paused due to the COVID19 pandemic) and adhered to the tenets of the Declaration of Helsinki. All patients provided written informed consent to treatment and participate in this study. Informed consent was obtained to publish information/images in an online open access publication.

### The study group

One hundred patients with blepharoptosis who met the inclusion criteria were scheduled to be enrolled in the study (the calculated statistical alpha 1 (type I) error was 5%^29,30^). Inclusion criteria were: (1) men and women of all races over the age of 18, (2) one or both eyes with moderate to severe upper eyelid ptosis (MRD1 < 2 mm or the difference in MRD1 of at least of 3 mm comparing to the contralateral eye), (3) at least “fair” function of the levator palpebrae superioris muscle (> 4 mm)^6,31^, (4) positive or negative test result with 10% phenylephrine administered topically into the conjunctival sac, (5) no prior ptosis surgery on the eyelids, and (6) no concomittant neurological disorders. Detailed medical histories of previous and concomittant diseases were collected with special attention to their etiology, duration, method and efficacy of treatment. Thereafter, a full ophthalmic examination was performed as previously described elsewhere^2,5^. The etiology of the blepharoptosis, the function of the levator muscle of the upper eyelid, and the degree of upper eyelid ptosis in mm (based on MRD1 measurements before and 10 min after the 10% phenylephrine eyedrops) were evaluated in a sitting position in each patient preoperatively^12,18,32^. The upper eyelids of all patients enrolled in the study were operated on by transconjunctival resection of the Mueller’s muscle and upper eyelid tarsus according to the algorithm:

*Algorithm:* 4 mm Mueller’s muscle transconjunctival resection to correct 1 mm ptosis of the upper eyelid (depending on a test with 10% phenylephrine: 3–12 mm) ± tarsal plate resection: 1 mm for every 1 mm of ptosis to correct residual ptosis after a phenylephrine test, but leaving a minimum of 4 mm upper tarsus intact.

**Figure 1:**
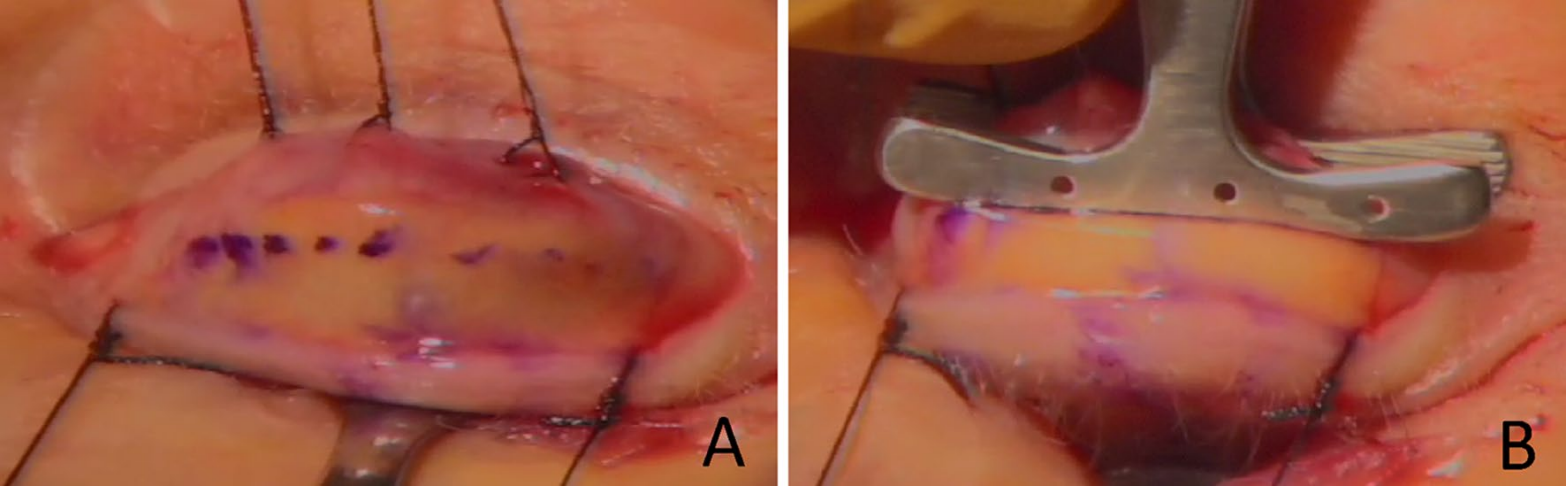
Intraoperative picture showing marked amount of tissues of the upper eyelid to be resected. (**A**) with traction sutures on the Mueller’s muscle. (**B**) with Putermann clamp on the Mueller’s muscle and part of the upper tarsus.

All surgeries were performed by a single surgeon (IN-G) under a microscope and with local anaesthesia. Patients were sterile prepped and draped. The upper eyelid landmarks (the center of the pupil and the limbus of the cornea from the nose and temples) were marked on the edge of the upper eyelid. Local anesthesia with 2% Xylocaine with adrenaline (1: 100 000) was infiltrated. Traction sutures on the edge of the upper eyelid were placed. The upper eyelid was inverted on the Desmaress retractor. The range of the Mueller’s muscle resection and the tarsus of the upper eyelid was marked with a skin marker. Traction sutures on the Mueller muscle (eg Mersilk 6–0) were placed (Fig. 1A). Three absorbable mattress sutures (Vicryl 6–0) per upper eyelid tarsus were placed about 1 mm below the Putermann clamp (Fig. 1B).

The determined amount of the conjunctiva, and the Mueller’s muscle, and the appropriate amount of the upper eyelid tarsus on the edge of the Putermann clamp was cut off with a scalpel (blade No. 11). Bleeding vessels were cauterized. Absorbable continuous sutures 8–0 were placed on the conjunctiva. A bandage contact lens was placed on the cornea. Antibiotic drops were instilled. An eyepad was placed on the eyelid.

In order to assess functional and cosmetic effects, photographic documentation was made during the preoperative visit, on the 7th day after the procedure, 6 months after the procedure, and during the evaluation of any complications. Outcomes were assessed on the basis of MRD1 change, symmetry, cosmetic effect, and analysis of complications. The criteria of full success was ptosis correction with intereyelid symmetry within 1 mm.

### The statistical analysis

Statistical analysis was performed using SPSS software (IBM Corp. Released 2012. IBM SPSS Statistics for Windows, Version 21.0. Armonk, NY: IBM Corp, USA). For measurable features, the normality of the distribution of analyzed parameters was evaluated using the Shapiro–Wilk test. The Wilcoxon pair order test was used to compare the two dependent groups. The Mann–Whitney U test was used to compare the two independent groups. For more than two independent groups, the Kruskal–Wallis test was used. A significance level of *p* < 0.05 was considered significant^29,30^.

## Results

**Figure 2:**
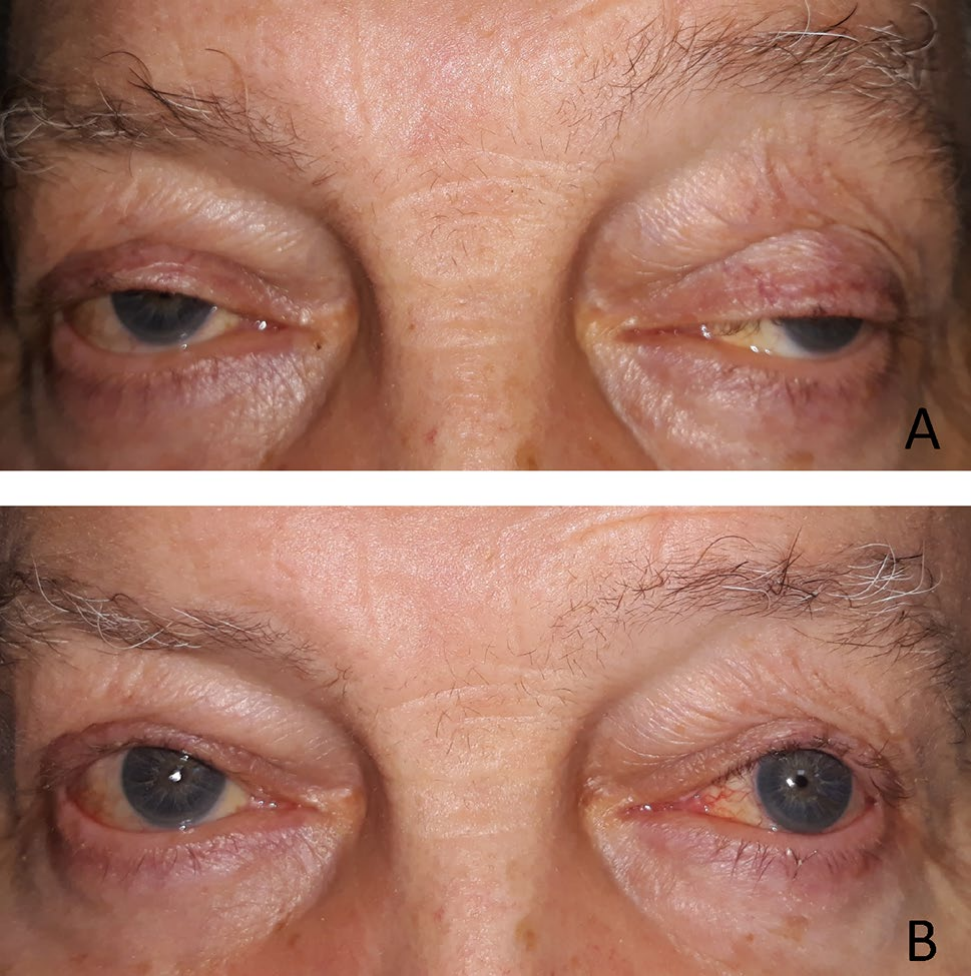
71-year-old patient after ptosis surgery in the right eye (8 mm of Mueller’s muscle resection and 2 mm of the tarsus) and the left eye (8 mm of Mueller’s muscle resection and 3.5 mm of the tarsus) at 6-month follow-up. (**A**) Before surgery. (**B**) The same patient at 6-month follow-up.

The study group included: 118 eyes of 81 patients, aged 69 (range: 47–87) years. Fifty-eight surgeries were performed unilaterally, 30 bilaterally (52 ptosis of the right eye and 66 of the left eye). The ptosis etiology was as follow: 97 eyes of 60 patients had involutional ptosis, 11 patients had congenital ptosis,7 patients had a history of trauma, and 3 patients had anophthalmia related ptosis. Follow-up assessments ranged from 6 to 32 months (mean 18 ± 5). Eyelid ptosis averaged 4.3 ± 2.6 mm (ranged 3–8 mm) before surgery. The function of the upper eyelid levator was 10.2 ± 3.4 (ranged 4–17) mm before surgery. Ten minutes after administration of 10% phenylephrine eyedrops, lifting of the upper eyelid by an average of 1.8 ± 0.7(ranged 0–3) mm was noted. Ptosis was corrected with the use of the algorhitm mentioned above in all patients in the study group (100%) (*p* < 0.001) (Fig. 2A,B). Full success with a mean MRD1 value > 3 and < 5 and intereyelid symmetry within 1 mm was noted in 109 out of 118 cases (92.4%). The mean MRD1 change was 3.9 ± 0.6 mm after surgery (*p* < 0.001). On average, 8.5 ± 0.8 (8–10) mm of Mueller’s muscle and 2.2 ± 0.9 (1–5) mm of the upper eyelid tarsus were removed surgically. Complications occurred in 9 out of 118 (7.6%) procedures. These were: (1) keratitis in 1 out of 118 (0.85%) cases, (2) undercorrection and asymmetry of > 1 mm in 6 out of 118 (5%) cases (which was 43% (3/7) of patients with traumatic ptosis and 27% (3/11) of patients with congenital ptosis), (3) visible change in the contour of the upper eyelid in 2 cases (1.7%). In 23% of patients, indications for the correction of dermatochalasis of the upper eyelid were noted. The mean operation time was 21 ± 7 min (range: 13–27).

## Discussion

Our study confirms that moderate to severe upper eyelid ptosis with at least “fair” function of the levetor muscle may be successfully corrected using a posterior approach. Putterman and Utrist introduce a 8.25 mm Mueller’s muscle resection transconjunctival approach as the way to treat mild upper eyelid ptosis^10^. Fasanella and Servat introduce and prove 3 mm Mueller’s muscle resection and 1-to-1 tarsectomy as the effective way to treat mild upper eyelid ptosis with a negative phenylephrine eyedrops result^11^. Further studies assess various modifications of previously established procedures in order to achive better results in a wider range of patients with blepharoptosis^23–26^. Weinstein and Buerger suggest 8 mm of Muellers muscle transconjunctival resection to correct 2 mm of ptosis^22^. Zauberman et al. suggest that removal of more than 8–9 mm of the Mueller’s muscle does not provide additional eyelid elevation but they do not correlate it with phenylephrine test results^32^. Cohen et al. do not routinely use phenylephrine response as a criterion for patient selection^33^. Interestingly the study of Ben Simon et al. reveals underestimation of phenylephrine test results in 40% of postoperative ptosis correction^12^. Contrary to these, Ayala et al. report excellent results using 10, 8, and 6 mm resections for 2, 1.5, and 1 mm (respectively) ptosis cases. Werb described the excision of a small amount of tarsus with Mueller’s muscle to enhance manipulation of the posterior lamella during dissection^25^. Perry et al. combine tranconjunctival Mueller’s muscle resection with a tarsectomy of maximum of 2.5 mm to correct mild to moderate upper eyelid ptosis with good function of the levator muscle^13^. Samimi et al. in 2013 reported good outcomes for their modified Fasanella-Servat procedure that includes tarsectomy, using a ratio of 2 mm of tarsus removed for every millimeter of desired eyelid elevation for the correction of minimal amounts of ptosis^26^. Georgescu et al. satisfactorily use Müller’s muscle conjunctival resection for blepharoptosis repair in 3 patients with poor to “fair” levator function. In 2020 Sveeney et al. and in 2021 Nacaroglu et al. report that Muller's muscle conjunctival resection may be a good option for the correction of severe involutional aponeurotic ptosis. Our study question was: if it is possible to correct moderate to severe upper eyelid ptosis using a posterior approach, which algorhitm should be used to achieve satisfactory outcomes?.

Studies of other authors ^10,19,23^ and our observations led us believe that the removal of 4 mm of the Mueller’s muscle results in about 1 mm elevation of the upper eyelid. Our study shows that the algorithm proposed and surgical technique used allows for the successful correction of moderate to severe ptosis in adults using a posterior approach, provided that the preoperative phenylephrine test result, Mueller’s muscle condition and weight and tarsus height are correlated. Our suggestion is: for ptosis correction of 5 mm in patients with at least 4 mm of levator function and elevation of the eyelid by 2 mm after instillation of phenylephrine eyedrops, 8 mm of the Mueller’s muscle and 3 mm of the upper tarsus should be resected. If the instillation of 10% phenylephrine eyedrops in the same patient resultes in the elevation of the upper eyelid by 1 mm – 4 mm of the Mueller’s muscle then 4 mm of the upper tarsus should be resected, provided that there is enough of the tarsus, which lets you leave at least 4 mm of the upper tarsus intact. According to our algorhitm, anatomical studies ^14,15^ and the surgical technique used, a maximal ptosis correction of about 7–8 mm is possible.

Our study has some limitations: the majority of the patients had involutional ptosis, thus a reliable comparative analyses between groups with different etiologies of blepharoptosis was not possible. Particular care should be taken when qualifying patients with ptosis due to anophthalmic socket. Although we obtained satisfactory results in our 3 patients, it should be remembered that the proposed method can lead to shortening of the posterior lamella, thus it cannot be the first-line method of treatment in patients with ptosis in anopththalmic socket, especially with shortened fornices ^33^. Nonetheless the algorithm and the surgical technique used allowed for the correction of even severe ptosis with only “fair” function of the upper eyelid levator and a negative 10% phenylephrine test result in about 15 min. Predictable, repeatable functional and cosmetic effects were achieved in cases of involutional ptosis in adults. Complications were rare and were noted after 7.6% of the surgical procedures. Keratitis in 1 patient was related to a bandage contact lens worn for too long stemming from the coronavirus pandemic. Undercorrection and asymmetry of > 1 mm in 6 out of 118 (5%) cases occurred in no patients with involutional ptosis and seems to be determined by anatomical changes of the upper eyelid in cases of traumatic or congenital ptosis ^33^. Visible change in the contour of the upper eyelid with residual nasal ptosis in 2 cases (1.7%) was related to inadequate Putterman clamp placement during surgery.

## Conclusion

Ptosis correction using a transconjunctival approach is a fast and effective method of treating moderate to severe upper eyelid ptosis with a low complication rate. The suggested method allowed for the correction of even severe ptosis with only “fair” function of the upper eyelid levator and a negative 10% phenylephrine test result. The most common complication was undercorrection, observed in patients with congenital and post-traumatic upper eyelid ptosis, which seems to be determined by anatomical changes of the upper eyelid and can be responsible for the suboptimal results in these groups. The algorithm used allows surgeons to achieve predictable, repeatable, functional and cosmetic outcomes as well as excellent symmetry, especially in adult patients with involutional ptosis of the upper eyelid. Our outcomes were encouraging but further comparative research using a larger sample is needed to draw detailed conclusions and show dependencies.

## Consent to participate

The photographs in the manuscript were taken by first author (IN-G).

## Data Availability

The datasets used and/or analysed during the current study are available from the corresponding author upon request.
